# Cervical Cancer Screening in South Florida Veteran Population, 2014 to 2020: Cytology and High-Risk Human Papillomavirus Correlation and Epidemiology

**DOI:** 10.7759/cureus.17247

**Published:** 2021-08-17

**Authors:** Lee B Syler, Corinne L Stobaugh, Philip R Foulis, George T Carlton, Lauren A DeLand, Andrew A Borkowski

**Affiliations:** 1 Pathology, University of South Florida, Tampa, USA; 2 Pathology and Laboratory Medicine, James A. Haley Veterans' Hospital, Tampa, USA; 3 Nursing, James A. Haley Veterans' Hospital, Tampa, USA

**Keywords:** human papillomavirus (hpv), hpv testing, cervical cancer screening, cervical cytology, veteran affairs, cervical cancer, hpv epidemiology, veteran women and hpv, hpv infection, papanicolaou

## Abstract

Objective

This project aims to use our robust women's health patient data to analyze the correlation between cytology and high-risk human papillomavirus (Hr-HPV) testing, study the performance of Hr-HPV testing for detecting cytology lesions, and examine epidemiologic measures of human papillomavirus (HPV) infections in the women's veteran population.

Methods

We collected patient data from 2014 to 2020 from our computerized patient record system. We performed HPV assays using the cobas® 4800 system (Roche Diagnostics, Basel, Switzerland). The cobas HPV assay detects HPV 16, HPV 18, and 12 other HPV types (31, 33, 35, 39, 45, 51, 56, 58, 59, 66, and 68). We organized cytology results and Hr-HPV assays with Microsoft Access and Microsoft Excel (Microsoft Corporation, Washington, USA) for analysis.

Results

A total of 9437 cervical specimens were co-tested. High-grade cytology lesions - high-grade intraepithelial lesion (HSIL) or higher and atypical squamous cells, cannot exclude HSIL (ASC-H) - were overwhelmingly positive for Hr-HPV (94.1% and 87.2%, respectively). Low-grade cytology lesions - low-grade squamous intraepithelial lesion ((LSIL) and atypical squamous cells of undetermined significance (ASC-US) - were positive for Hr-HPV in lower percentages (72.6% and 54.9%, respectively). Hr-HPV testing had a sensitivity of 91.3%, a specificity of 93.1%, a positive predictive value of 16.4%, and a negative predictive value of 99.8% for detecting high-grade cytology lesions. Hr-HPV testing had a lower performance for detecting low-grade cytology lesions. Ten cases had high-grade cytology and negative Hr-HPV test. Out of 10 such patients, nine showed no dysplasia (six) or low-grade dysplasia (three) on subsequent biopsy. Overall, 14.4% of tests were positive for Hr-HPV. The highest positive Hr-HPV test rates were in the third and eighth decades of life, 25.1% and 22.0%, respectively. However, the eighth decade consisted of a small sample of only 50 women. In women over 30 years of age with Hr-HPV infections, HPV types 16 and 18 were present in 11.7% and 6.4% of tests, respectively. Other HPV types were present in 82.3% of tests.

Conclusions

Hr-HPV testing has a high performance in detecting high-grade cytology lesions and a lower performance for detecting low-grade cytology lesions. However, studies show that LSIL rarely progresses to cervical intraepithelial neoplasia grade 3 or higher (CIN3+), suggesting minimal to no impact on cervical cancer screening. We believe our findings are in accordance with recent studies and affirm the guidelines that recommend primary Hr-HPV testing as the preferred screening method. The percentage of positive Hr-HPV tests and rates for age and HPV types 16 and 18 in our women’s veteran population suggest similar HPV prevalence to that of the general US population.

## Introduction

Human papillomavirus (HPV) is one of the most potent carcinogens in humans, with almost 5% of all new cancers diagnosed worldwide attributable to HPV [1]. These cancers include cervical, anal, vaginal, penile, vulvar, and oropharyngeal. Almost all cervical cancers are HPV-related [1]. HPV is estimated to be the most common sexually transmitted disease (STD) infection in the United States (US) [2]. Although broad-spread screening practices and vaccination have significantly decreased the incidence and mortality of cervical cancer in the US, an estimated 14,480 new cervical cancer diagnoses and 4,290 deaths will occur in 2021 [3].

HPV infects epithelial cells, promoting cellular proliferation, blocking apoptosis, and evading the immune system [4,5]. Persistent infection is necessary for the development of cervical intraepithelial neoplasia (CIN), and the probability of complete clearance is dependent on the duration of infection [6]. Important factors that determine infection progression are the HPV genotype and immunosuppression [7]. Their carcinogenic effects have classified HPV genotypes as high-risk HPVs (Hr-HPV) and low-risk HPVs (Lr-HPV). Of the Hr-HPVs, types 16 and 18 are the most persistent and carcinogenic [8]. Thankfully, the vast majority of HPV infections are cleared by the immune system despite immune evasion mechanisms of the virus; about 70% within one year and about 90% within two years [9].

Since the 1950s, for most of this period, the basis of cervical cancer screening has relied on the revolutionary Papanicolaou test and, later, liquid-based cytology. As cervical screening technologies evolved and knowledge of the natural history of cervical cancer grew, so did the recommendations for screening and management.

One of the most substantial changes in screening and management guidelines was the incorporation of Hr-HPV molecular testing into screening protocols for cervical cancer by the American Society for Colposcopy and Cervical Pathology (ASCCP) in 2012 [10]. At that time, they did not recommend primary Hr-HPV testing (only Hr-HPV testing without cytology) for screening due to concerns of specificity and possible excess treatment of non-neoplastic HPV lesions.

In 2015, after growing evidence of the high performance of primary Hr-HPV testing [11-14] and the FDA approval of an Hr-HPV assay for primary Hr-HPV screening, the ASCCP published an Interim Clinical Guidance for the use of primary Hr-HPV testing for cervical cancer screening [15].

In 2020, the American Cancer Society (ASC) updated the cervical cancer screening guidelines. They recommended primary HPV testing as the preferred screening method, with co-testing and cytology alone being acceptable [16]. They also mentioned that the guidelines should be transitional towards primary HPV testing and that co-testing and cytology alone should gradually phase out. The current FDA-approved HPV assays for primary HPV screening are the cobas® HPV test (Roche Diagnostics, Basel, Switzerland) and The BD Onclarity™ HPV Assay (BD Diagnostics, Franklin Lakes, New Jersey, US). The FDA approves these and other assays for co-testing [16].

During the last decades, the Veterans Health Administration (VHA) has seen a significant increase in its women patient population. The growing numbers of women participating in the US military have made this subgroup the fastest growing subgroup of US veterans [17]. The VHA commits to delivering comprehensive primary care for women. A designated women's health primary care provider (WH-PCP), who leads a Women's Health Patient Align Care Team (WH-PACT), provides this care. The WH-PCP and WH-PACT must have sufficient training and expertise to care for women veterans [18].

Although recent cervical screening guidelines shift towards primary HPV screening as the main method for cervical cancer screening, we use co-testing at James A. Haley Veterans Affairs Hospital. Co-testing provides an excellent opportunity for comparing and correlating cytology and Hr-HPV testing results. Studies that compare cytology testing to Hr-HPV testing in the US women's veteran population are lacking. As the gradual shift towards primary Hr-HPV screening occurs, we believe more studies will compile in favor of the performance of Hr-HPV testing in different scenarios.

Female veterans have a higher prevalence of interpersonal trauma due to their military service, affecting their healthcare needs [19-21]. Although it is not yet clear if veteran women have a higher prevalence of HPV infection and cervical cancer than the general population, some studies suggest that this may be the case [22-24]. Although researchers have performed studies on HPV prevalence in the general population of the US [25-27], these studies are lacking in the US women veteran population. More studies on cervical cancer and HPV prevalence affecting the US women's veteran population are needed.

This project aims to use our robust women's veteran health data to analyze the correlation between Hr-HPV testing and cervical cytology. In doing so, we measure the performance of Hr-HPV testing for the detection of high-grade versus low-grade lesions diagnosed by cytology within this specific population. We also calculate the percent of positive Hr-HPV tests among US veteran women, a population in which these studies are scant.

(This article was previously posted as a preprint on the medRxiv preprint server on June 14, 2021.)

## Materials and methods

A total of 9,437 cervical specimens of women veterans were co-tested by Hr-HPV testing and cytology from 2014 through 2020.

The Hr-HPV testing was done through the cobas HPV Test using the Roche cobas 4800 System. The cobas HPV Test detects HPV 16, HPV 18, and 12 other HPV types (31, 33, 35, 39, 45, 51, 52, 56, 58, 59, 66, and 68). 

We divided cytology results into two main diagnostic categories: high-grade cytology lesions, including high-grade intraepithelial lesion (HSIL) or higher and Atypical squamous cells, cannot exclude HSIL (ASC-H), and low-grade cytology lesions, including low-grade squamous intraepithelial lesion ((LSIL) and atypical squamous cells of undetermined significance (ASC-US). Results of cytology and Hr-HPV assays were sorted, organized, and analyzed using Microsoft Access and Microsoft Excel (Microsoft Corporation, Washington, US).

We calculated percentages of Hr-HPV positive cases for each cytologic diagnostic category. We evaluate sensitivity, specificity, positive predictive value, and negative predictive value of the Hr-HPV test for detecting each cytologic diagnostic category. We further analyzed cases that showed high-grade cytology but resulted in a negative Hr-HPV test looking for common findings and possible causes of discrepancy. 

We calculated the total percentage of Hr-HPV positive cases within the population. We divided women by age groups (20-29, 30-39, 40-49, 50-59, 60-69, 70-79, and >80) and calculated the percentages of positive Hr-HPV in each age group. In women over age 30 with Hr-HPV infections, we determined the rate of positive tests for HPV types 16, 18, and other types precisely. We did not determine the specific rates for Hr-HPV types in women under age 30 because we do not report specific Hr-HPV type results in this younger age group. We only report Hr-HPV type results in women over age 30 with a cytology result of ASC-US, LSIL, or higher.

## Results

**Figure 1:**
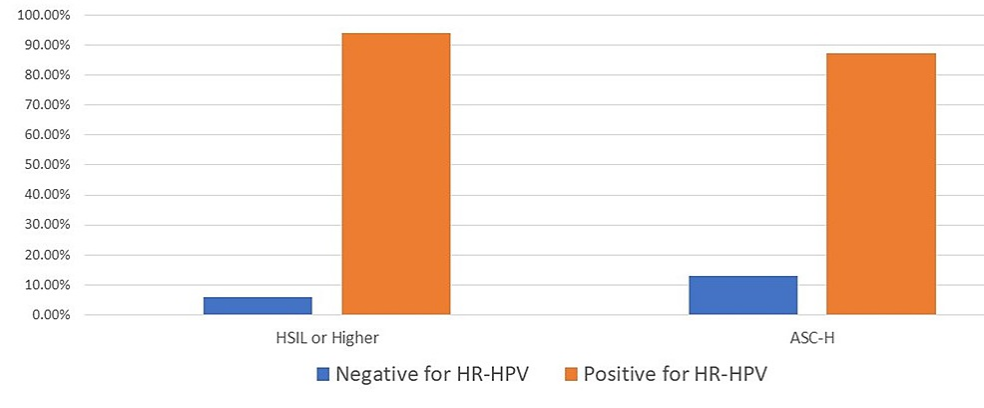
Hr-HPV results in high-grade cytology lesions Hr-HPV: High-risk human papillomavirus; HSIL: High-grade intraepithelial lesion; ASC-H: Atypical squamous cells, cannot exclude HSIL

Of the 9,437 cervical specimens of women veterans co-tested during 2014-2020, the Hr-HPV test results for high-grade cytology lesions (HSIL or higher and ASC-H) were overwhelmingly positive for Hr-HPV (94.1% and 87.2%, respectively) (Figure1).

**Figure 2:**
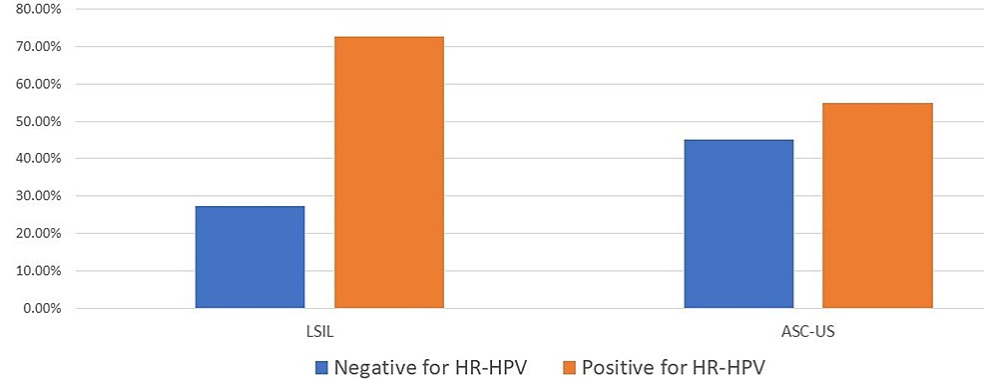
Hr-HPV results in low-grade cytology lesions Hr-HPV: High-risk human papillomavirus; LSIL: Low-grade squamous intraepithelial lesion; ASC-US: Atypical squamous cells of undetermined significance

Low-grade cytology lesions (LSIL and ASC-US) were positive for Hr-HPV in lower percentages (72.6% and 54.9%, respectively) (Figure 2).

**Figure 3:**
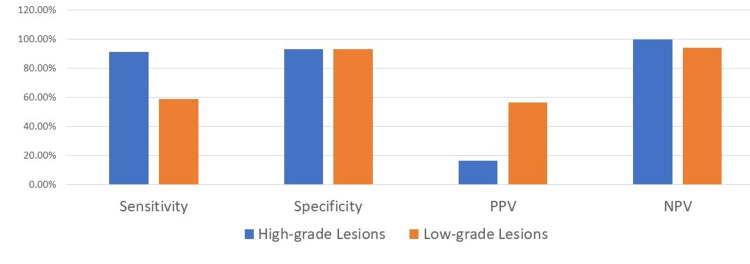
Performance of Hr-HPV testing on high-grade vs low-grade cytology lesions Hr-HPV: High-risk human papillomavirus; PPV: Positive predictive value; NPV: Negative predictive value

Hr-HPV testing had a sensitivity of 91.3%, a specificity of 93.1%, a positive predictive value (PPV) of 16.4%, and a negative predictive value (NPV) of 99.8% for detecting high-grade cytology lesions. However, it had a lower sensitivity of 58.9%, a specificity of 93.1%, a PPV of 56.2%, and an NPV of 93.8% for detecting low-grade cytology lesions. Figure 3 compares these validity measures of Hr-HPV testing for high-grade versus low-grade lesions diagnosed by cytology.

A review of medical records of patients with high-risk lesions on cytology and negative Hr-HPV showed that nine out of 10 patients had either no dysplasia (six) or low-grade dysplasia (three) on subsequent biopsy. Two of these cases showed microglandular hyperplasia; one of these simultaneously with low-grade dysplasia. The remaining case was diagnosed cytologically as ASC-H but was lost to follow-up and did not undergo biopsy. Cytology review of most of these cases identified significant background inflammation complicating cytologic interpretation.

Out of the 9437 cervical specimens, 1359 cases were positive for Hr-HPV, constituting 14.4% (Table 1).

**Table 1:** Hr-HPV results of co-tests 2014-2020

Hr-HPV results of co-tests 2014-2020	Number/total	Percentage of total
Positive Hr-HPV	1359/9437	14.40%
Negative Hr-HPV	8078/9437	85.60%

Description of age group-specific positive Hr-HPV percentages is in Table 2. The highest positive Hr-HPV test rates were in the third and eighth decades of life, 25% and 22%, respectively.

**Table 2:** Age group-specific positive Hr-HPV percentages

Age group	Total number (% of total population)	Number of positive Hr-HPV (% of total positives)	Percentage of positives for age group
20-29	1325 (14.0%)	333 (24.5%)	25.1%
30-39	2639 (28.0%)	440 (32.4%)	16.7%
40-49	2274 (24.1%)	254 (18.7%)	11.2%
50-59	2127 (22.5%)	223(16.4%)	10.5%
60-69	1015 (10.8%)	98 (7.2%)	9.7%
70-79	50 (0.5%)	11 (0.8%)	22.0%
>80	7 (0.1%)	0 (0.0%)	0.0%
Total	9473 (100.0%)	1359 (100%)	14.4%

In women over age 30, 1036 were positive for HR-HPV. Of these cases, 121 contained HPV type 16 as a single or coinfection (11.7%), 66 contained HPV type 18 (6.4%) as a single or coinfection, and 852 contained Hr-HPV types other than 16 and 18 (82.3%) (Table 3).

**Table 3:** Hr-HPV infection percentages by types in women over age 30 Hr-HPV: High-risk human papillomavirus

Hr-HPV infection type in women over age 30	Number/total	Percentage of total
HPV type 16 as single or coinfection	121/1036	11.70%
HPV type 18 as single or coinfection	66/1036	6.40%
Hr-HPV types other than 16 and 18	852/1036	82.30%

## Discussion

Some of the most extensive studies worldwide have demonstrated the superior performance of primary Hr-HPV screening compared to cytology screening alone [11-14]. These studies have shown that incidence risks for cervical intraepithelial neoplasia grade 3 and higher (CIN3+) are higher in women screened with cytology alone when compared to those screened with primary Hr-HPV testing. But what about co-testing? Wouldn't the combination of both screening strategies add to higher performance? Gage et al. [12] answered this question in their study of close to 1 million screened women. They showed that the three-year risk for developing CIN3 and cancer after a negative Hr-HPV test was lower when compared to the five-year risk for developing CIN3 and cancer after a negative co-test. The authors suggested that the co-test derived most of its reassurance from the Hr-HPV testing portion.

In their study results from the ATHENA study, Wright et al. [13] also demonstrated an increased risk of CIN3+ in women screened with cytology alone than those screened by Hr-HPV testing. They showed that Hr-HPV testing had a sensitivity of 76.1% for detecting CIN3+, higher than the 47.8% and 61.7% for cytology and co-testing, respectively.

Although we did not follow our patients to calculate incidence risks of CIN3 or cancer in our population, our results show a high positive correlation between high-grade cytology lesions and positive Hr-HPV testing results. The majority of high-grade cytology lesions were positive for Hr-HPV (94.1% of HSIL and higher, 87.2% for ASC-H). Hr-HPV testing was highly sensitive and specific for detecting high-grade cytology lesions, 91.3% and 93.1%, respectively. It also had an NPV of 99.8%, which gives high reassurance to those women with a negative Hr-HPV result. A low PPV of 16.4% is obvious since HPV types 16 and 18, together, have a three-year cumulative incidence risk (CIR) of only 21.16% for developing CIN3+, and the other Hr-HPVs have a three-year CIR of 5.4% for developing CIN3+, according to the ATHENA study [13]. This means that not all Hr-HPV infections will progress to high-grade dysplasia, hence the low PPV.

When reviewing positive high-grade cytology lesions that were negative for Hr-HPV testing, we found that the majority had significant background inflammation complicating cytologic interpretation. On subsequent biopsy, nine out of 10 were either negative for dysplasia (six) or had low-grade dysplasia (three). Two of the revised biopsies showed microglandular hyperplasia, one of them with simultaneous low-grade dysplasia. Microglandular hyperplasia is a benign alteration of the endocervical epithelium in which the endocervical cells can show reactive changes. These changes can be confused with a wide range of differential diagnoses during cytological interpretation, including LSIL, HSIL, AIS, and invasive cancer [28]. These are only some examples of situations in which cytological interpretation can be equivocal.

A concerning finding in our study is the low sensitivity of Hr-HPV testing for detecting low-grade cytology lesions. One cannot help but ask: What is the impact of missing low-grade lesions? LSIL diagnosed by cytology can be caused by Hr-HPV types or Lr-HPV types. A study from the Netherlands showed that, over four years, all women with LSIL and a negative Hr-HPV test regressed to normal cytology, as did 85% of those with HSIL and a negative Hr-HPV test [29]. Another study done in Brazil showed that more than 90% of women with a cytology result of LSIL regressed within 24 months [30]. These studies suggest that not detecting a percentage of low-grade cytology lesions through primary Hr-HPV testing would not significantly impact screening for cervical cancer.

Our results show that 14.4% of all Hr-HPV tests were positive in our women's veteran population. Our literature search for the overall prevalence of Hr-HPV types in the US general population found variable results. A study carried out by Monsonego et al. used extensive data from the ATHENA trial with over 40,000 women screened in 23 states. They showed the lowest overall Hr-HPV prevalence, 13.4% [26], which is very close to our calculations of the total percentage of positive Hr-HPV tests. Another study showed a similar prevalence of 15.2% [24], and another showed a higher prevalence of 20.4% [26] among the general US female population. These numbers suggest that, in the women's veteran population that we serve, Hr-HPV prevalence is not higher than that of the general US population; in fact, it suggests similarity.

When analyzing age-specific rates of Hr-HPV positive tests, we found them very similar to the rates of the women’s general public calculated from the ATHENA study results (See Table 4) [13]. Our 20-29 age group had the highest Hr-HPV positive rate, similar to their 25-29 age group, 25% versus 21%, respectively. Our data show a second spike in positive rates in the eighth decade of life (22%). However, our number of cases is small in this age group, only 50 patients. Wright et al. did not further subdivide their age groups past 50; hence we could not compare to see if a similar spike exists in the general population.

**Table 4:** Comparison of age-specific Hr-HPV positive rates between our US women's veteran population and that of the general US population calculated by Wright et al.

Our US women's veteran population		General US population from ATHENA Study results (Wright et al. [13])	
Age group	Hr-HPV positive test (% of age group)	Age group	HR-HPV positive (% of age group)
20-29	25.1%	25-29	21.1%
30-39	16.7%	30-39	11.6%
40-49	11.2%	40-49	7.1%
50-59	10.5%	>50	6.0%
60-69	9.7%		
70-79	22.0%		
>80	0.0%		

When comparing the percentage of HPV types 16 and 18 positive cases between our population and that of the general US female population analyzed from the ATHENA study data by Monsonego et al., HPV type 16 had a lower percentage in our population, 11.7% versus 18.9%, and HPV type 18 had a similar rate in our population, 6.4% versus 7.2%. Our percentages fall very close to that of the general US female public in a study by Dunne et al. [25], it is 11.7% versus 9.9% for HPV type 16 and 6.4% versus 5.3% for HPV type 18 out of all positive Hr-HPV cases.

## Conclusions

According to our data, Hr-HPV testing shows high-performance measures for detecting high-grade cytology lesions in our women's veteran population. However, it will miss a significant number of low-grade lesions. Studies have shown that LSIL rarely progresses to CIN3+, primarily when Lr-HPV types cause it. These studies suggest that not detecting a percentage of these lesions has minimal to no impact on cervical cancer screening. We believe our findings are in accordance with recent studies and guidelines that recommend primary Hr-HPV testing as the preferred screening method. The total percentage of positive Hr-HPV tests, rates for age, and rates for HPV types 16 and 18 in our women's veteran population suggest similar HPV prevalence to that of the general US population.

## References

[1] de Martel C, Ferlay J, Franceschi S, Vignat J, Bray F, Forman D, Plummer M (2012). Global burden of cancers attributable to infections in 2008: a review and synthetic analysis. Lancet Oncol.

[2] Kreisel KM, Spicknall IH, Gargano JW (2021). Sexually transmitted infections among US women and men: prevalence and incidence estimates, 2018. Sex Transm Dis.

[3] Siegel RL, Miller KD, Fuchs HE, Jemal A (2021). Cancer statistics, 2021. CA Cancer J Clin.

[4] Ghittoni R, Accardi R, Hasan U, Gheit T, Sylla B, Tommasino M (2010). The biological properties of E6 and E7 oncoproteins from human papillomaviruses. Virus Genes.

[5] Piersma SJ (2011). Immunosuppressive tumor microenvironment in cervical cancer patients. Cancer Microenviron.

[6] Rodríguez AC, Schiffman M, Herrero R (2010). Longitudinal study of human papillomavirus persistence and cervical intraepithelial neoplasia grade 2/3: critical role of duration of infection. J Natl Cancer Inst.

[7] IARC Working Group on the Evaluation of Carcinogenic Risks to Humans (2012). Biological agents. Volume 100 B. A review of human carcinogens. Monogr Eval Carcinog Risks Hum.

[8] Castle PE, Rodríguez AC, Burk RD (2009). Short term persistence of human papillomavirus and risk of cervical precancer and cancer: population based cohort study. BMJ.

[9] Ho GY, Bierman R, Beardsley L, Chang CJ, Burk RD (1998). Natural history of cervicovaginal papillomavirus infection in young women. N Engl J Med.

[10] Massad LS, Einstein MH, Huh WK (2013). 2012 updated consensus guidelines for the management of abnormal cervical cancer screening tests and cancer precursors. J Low Genit Tract Dis.

[11] Ronco G, Dillner J, Elfström KM (2014). Efficacy of HPV-based screening for prevention of invasive cervical cancer: follow-up of four European randomized controlled trials. Lancet.

[12] Gage JC, Schiffman M, Katki HA (2014). Reassurance against future risk of precancer and cancer conferred by a negative human papillomavirus test. J Natl Cancer Inst.

[13] Wright TC, Stoler MH, Behrens CM, Sharma A, Zhang G, Wright TL (2015). Primary cervical cancer screening with human papillomavirus: end of study results from the ATHENA study using HPV as the first-line screening test. Gynecol Oncol.

[14] Dillner J, Rebolj M, Birembaut P (2008). Long term predictive values of cytology and human papillomavirus testing in cervical cancer screening: joint European cohort study. BMJ.

[15] Huh WK, Ault KA, Chelmow D (2015). Use of primary high-risk human papillomavirus testing for cervical cancer screening: interim clinical guidance. Gynecol Oncol.

[16] Fontham ET, Wolf AM, Church TR (2020). Cervical cancer screening for individuals at average risk: 2020 guideline update from the American Cancer Society. CA Cancer J Clin.

[17] Frayne SM, Yu W, Yano EM, Ananth L, Iqbal S, Thrailkill A, Phibbs CS (2007). Gender and use of care: planning for tomorrow's Veterans Health Administration. J Womens Health (Larchmt).

[18] Department of Veterans Affairs (2017). Health care services for woman veterans.VHA handbook 1330.01(4). Administration, V.H., VHA Handbook 1330.01(4):.

[19] Zephyrin LC (2016). Reproductive health management for the care of women veterans. Obstet Gynecol.

[20] Zinzow HM, Grubaugh AL, Frueh BC, Magruder KM (2008). Sexual assault, mental health, and service use among male and female veterans seen in Veterans Affairs primary care clinics: a multi-site study. Psychiatry Res.

[21] Zinzow HM, Grubaugh AL, Monnier J, Suffoletta-Maierle S, Frueh BC (2007). Trauma among female veterans: a critical review. Trauma Violence Abuse.

[22] Sadler AG, Mengeling MA, Syrop CH, Torner JC, Booth BM (2011). Lifetime sexual assault and cervical cytologic abnormalities among military women. J Womens Health (Larchmt).

[23] Ollayos CW, Peterson M (2002). Relative risks for squamous intraepithelial lesions detected by the Papanicolaou test among Air Force and Army beneficiaries of the Military Health Care System. Mil Med.

[24] Goyal V, Mattocks KM, Sadler AG (2012). High-risk behavior and sexually transmitted infections among U.S. active duty servicewomen and veterans. J Womens Health (Larchmt).

[25] Dunne EF, Unger ER, Sternberg M, McQuillan G, Swan DC, Patel SS, Markowitz LE (2007). Prevalence of HPV infection among females in the United States. JAMA.

[26] McQuillan G, Kruszon-Moran D, Markowitz LE, Unger Unger, ER ER, Paulose-Ram R (2017). Prevalence of HPV in adults aged 18-69: United States, 2011-2014. NCHS Data Brief, no 280. National Center for Health Statistics.

[27] Monsonego J, Cox JT, Behrens C, Sandri M, Franco EL, Yap PS, Huh W (2015). Prevalence of high-risk human papilloma virus genotypes and associated risk of cervical precancerous lesions in a large U.S. screening population: data from the ATHENA trial. Gynecol Oncol.

[28] Cibas ES, Ducatman BS (2019). Cytology: diagnostic principles and clinical correlates, 5th edition. https://www.elsevier.com/books/cytology/cibas/978-0-323-63636-0.

[29] Nobbenhuis MA, Helmerhorst TJ, van den Brule AJ (2001). Cytological regression and clearance of high-risk human papillomavirus in women with an abnormal cervical smear. Lancet.

[30] Schlecht NF, Platt RW, Duarte-Franco E (2003). Human papillomavirus infection and time to progression and regression of cervical intraepithelial neoplasia. J Natl Cancer Inst.

